# Leaching Process of Weathered Crust Elution-Deposited Rare Earth Ore With Formate Salts

**DOI:** 10.3389/fchem.2020.598752

**Published:** 2020-12-03

**Authors:** Zhuo Chen, Zhenyue Zhang, Ruan Chi

**Affiliations:** ^1^School of XingFa Mining Engineering, Wuhan Institute of Technology, Wuhan, China; ^2^Key Laboratory for Green Chemical Process of Ministry of Education, Wuhan Institute of Technology, Wuhan, China

**Keywords:** weathered crust elution-deposited rare earth ore, formate salts, leaching process, rare earth, aluminum, swelling

## Abstract

To strengthen the rare earth leaching process and weaken the hydration of clay minerals for preventing landslides, it is of great importance to adopt a green and sustainable leaching agent in the industry. In this work, the leaching process of weathered crust elution-deposited rare earth ores with formate salts (ammonium formate, potassium formate, and sodium formate) was investigated. The effects of formate salts on the linear swelling ratio and zeta potential of the clay minerals were studied. The experimental results showed that ammonium formate could effectively recover the rare earth elements from weathered crust elution-deposited rare earth as well as inhibit the leaching of impurity aluminum. At room temperature, when the ammonium formate concentration was 1% wt, the leaching efficiencies of rare earth and aluminum were 87 and 37%, respectively. Compared with traditional inorganic ammonium salts, the inhibition effect of impurity aluminum was obvious. In addition, the results of the linear swelling ratio in the clay minerals showed that the inhibit ability of formate salts on the hydration of clay minerals enhanced with the increase of the formate concentration, and the order of the inhabitation on the clay minerals followed: 1% ammonium formate > 1.5% potassium formate > 2.5% sodium formate > distilled water. Based on the double layer theory, ammonium formate and potassium formate could effectively compress clay mineral particles to avoid water intake, which could increase the interaction between clay mineral particles and greatly reduce the electronegative property of the clay minerals, so as to effectively reduce the surface hydration of clay minerals to decrease the swelling of rare earth ore. The results of this experiment have important and practical significance in guiding the prevention of landslides, promoting the *in-situ* leaching technology, and effectively protecting the ecological environment in mining areas.

## Introduction

Medium and heavy rare earth elements are widely adopted in the material field of the high-tech and national defense industry which has led to the supplement growth of this strategic resource (Kynicky et al., 2012; Simandl, 2014). Weathered crust elution-deposited rare earth ores are rich in medium and heavy rare earth elements, which makes up for the shortage of medium and heavy rare earth elements in mineral-type rare earth minerals (Zhang et al., 2016).

These weathered crust elution-deposited rare earth ores are distributed in the southern part of China. The rare earth elements mainly exist as hydrated ions or hydroxyl hydrated ions which are adsorbed on the surface of the clay mineral in this kind of rare earth ore (He et al., 2016a). Based on the special characteristics of the weathered crust elution-deposited rare earth ore, conventional beneficiation technology cannot separate and enrich rare earth elements. Therefore, the rare earth elements are recovered under the ion exchange law with electrolytes. The leaching chemical reaction with mono valence salts is written as (Chi and Tian, 2006; Li, 2014; He et al., 2016b):

(1) $$\begin{array}{c} {\left[Al_{4}\left(Si_{4}O_{10}\right){\left(OH\right)}_{8}\right]}_{m}\cdot nRE_{\left(S\right)}^{3+}+{3nA}_{\left(aq\right)}^{+}⇌{\left[Al_{4}\left(Si_{4}O_{10}\right){\left(OH\right)}_{8}\right]}_{m}\cdot \\ {\left(A_{\left(aq\right)}^{+}\right)}_{3n\left(s\right)}+nRE_{\left(aq\right)}^{3+} \end{array}$$

where s represents the solid phase, and a represents the liquid phase.

After decades of development, ammonium chloride, and ammonium sulfate are mainly used as leaching agents to recover rare earth elements from the ore by the *in-situ* leaching technology in the industry at present.

**Figure 1:**
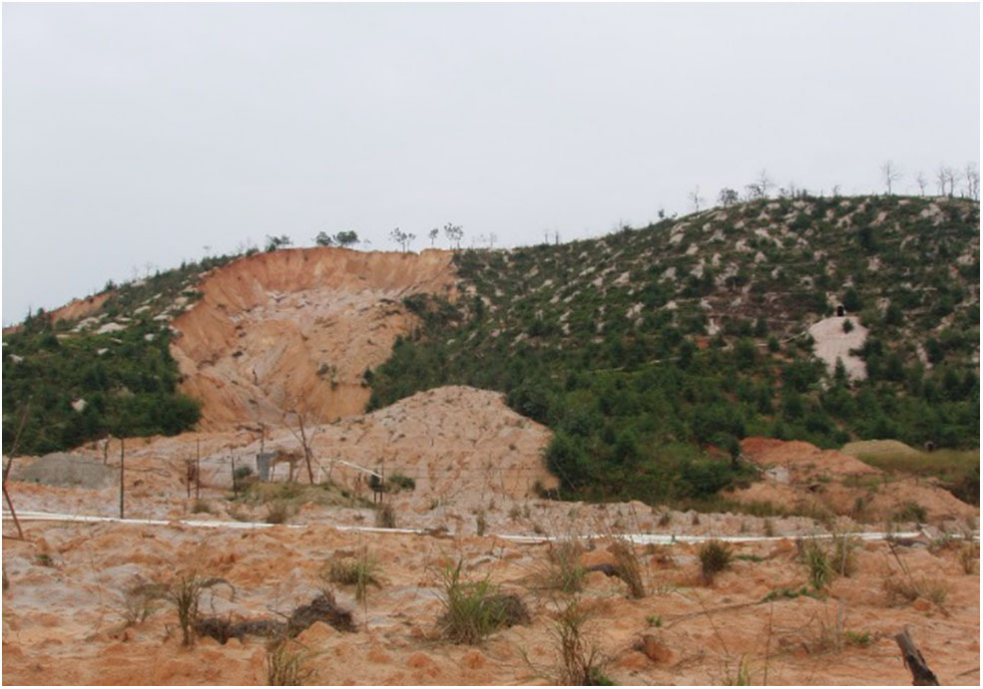
The landslide scene in the weathered crust elution-deposited rare earth ore.

However, there is normally high impurity content in lixivium which raises the cost of following industrial procedures. Besides, due to the improper injection of leaching agents, it would lead to the swelling of clay minerals under the physical and chemical function in the weathered crust elution-deposited rare earth ore body, which cause landslides and other geological disasters. An example landslide is shown in Figure 1. These problems would affect the mine safety and reduce the rare earth recovery efficiency.

To better improve the rare earth leaching efficiency and decrease the risks in the mining area, many experts have undertaken a great deal of research on the selection of new types of leaching agents which can effectively recover rare earth elements and reduce the swelling of clay minerals. Zhang et al. carried out research on the field of the swelling properties in clay minerals within the various layers of weathered crust elution-deposited ore bodies and found that the clay minerals in the humus layer were the most prone to expansion (Zhang et al., 2018a,b). Chen et al. found that magnesium salts can recover rare earth elements and inhibit the swelling of clay minerals effectively in the leaching process (Chen et al., 2018a,b, 2020). Norrish et al. found that with the decrease of electrolyte concentration in the solution, the intergranular spacing of the clay minerals would increase, which led to the swelling phenomenon (Norrish, 1954). Jiang et al. studied the inhibiting effect of sodium methyl silicate on shale expansion, and found that methyl silicate ions could adsorb on the edges and angles of clay mineral particles, making the hydrophilic surface of the clay mineral particles hydrophobic, thus preventing the water molecules from entering the interlayer of the clay mineral particles (Jiang et al., 2014). Ammonium acetate can readily exchange with exchangeable cations on the surface of clay minerals, and the molecular weight of acetic acid ions is small and electronegative, which can be absorbed between the clay mineral particles through electrostatic attraction and hydrogen bonds, thus reducing the tendency of clay minerals to absorb water (Erwan et al., 2011).

Although some achievements have been made in the development and research of new leaching agents to recover the rare earth elements effectively and environmentally, there are still some problems to be solved. Fomate salts can effectively improve the leaching rate of rare earth to a greater extent, while the inhibition on aluminum leaching by formate salts has been discovered. Meanwhile, the formate can promote the permeability of the leaching solution in the rare earth ore body. The purpose of this paper is to explore the leaching kinetic process of rare earth and aluminum from weathered crust elution-deposited rare earth ores with three formate salts (ammonium formate, potassium formate, and sodium formate), and to study the swelling properties of clay minerals during the leaching process. It could provide a new method to extract rare earth from ores with high efficiency, low consumption, and environmental protection, providing a theoretical basis for the industrial application of new leaching agents.

## Experiments

### Experimental Samples

The rare earth ore samples used in the experiments were collected from weathered crust elution-deposited rare earth ore in the Jiangxi Province, China. The main chemical composition of the rare earth ore was determined by x-ray fluorescence (Axios advanced, Panalytical B.V.), and the results of the rare earth ore sample is listed in Table 1.

**Table 1:** Main chemical composition of the rare earth ores (wt.%).

**Component**	**REO**	**Al_**2**_O_**3**_**	**MnO_**2**_**	**ZnO**	**CaO**	**MgO**	**K_**2**_O**	**SiO_**2**_**
Content	0.14	15.23	0.01	0.01	0.02	0.62	3.54	66.48
**Component**	**SO**_**3**_	**TiO**_**2**_	**Fe**_**2**_**O**_**3**_	**Rb**_**2**_**O**	**SrO**	**ZrO**_**2**_	**BaO**	**Loss**
Content	0.04	1.40	4.07	0.01	0.01	0.02	0.04	8.36

It can be seen from Table 1 that the main components are SiO_2_ and Al_2_O_3_, accounting for 66.48 and 15.23% in the rare earth ore. The content of rare earth in the ore sample is 0.14%. Aluminum is the main impurity in the leaching lixivium. The existence of impurities will increase the difficulty of rare earth recovery and reduce the purity of rare earth productions.

**Figure 2:**
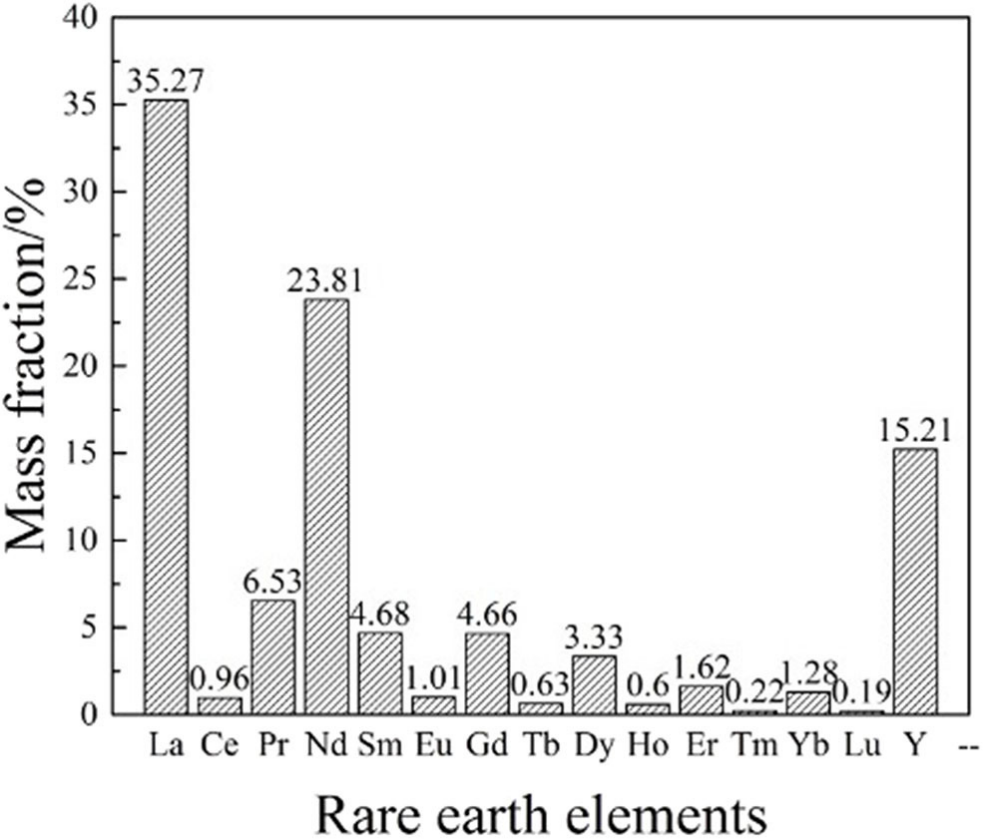
The rare earth partition of rare earth ores.

Besides, the distribution of weathered crust elution-deposited rare earth ore is one of the important indexes to measure its industrial value. The rare earth partition of exchangeable phase state in the ore sample was measured by inductively coupled plasma mass spectrometry (ICP-MS, Agilent 7700x, Agilent Technologies Inc.) and the result is shown in Figure 2.

It can be seen from Figure 2 that the total content of La_2_O_3_ and Nd_2_O_3_ account for 88.71% of the total amount of light rare earth and rare earth oxides, while the content of Y_2_O_3_ accounts for 45.63% of the medium heavy rare earth, indicating that the rare earth ore sample is mainly rich in heavy rare earth elements and has great commercial value.

Ammonium formate, potassium formate, sodium formate, and other chemical reagents used in the experiments were of analytical (AR) grade and were purchased from the Shanghai Chemical Reagent Company (Shanghai, China).

### Experimental Device and Method

#### The Leaching Experiments of Weathered Crust Elution-Deposited Rare Earth Ore

**Figure 3:**
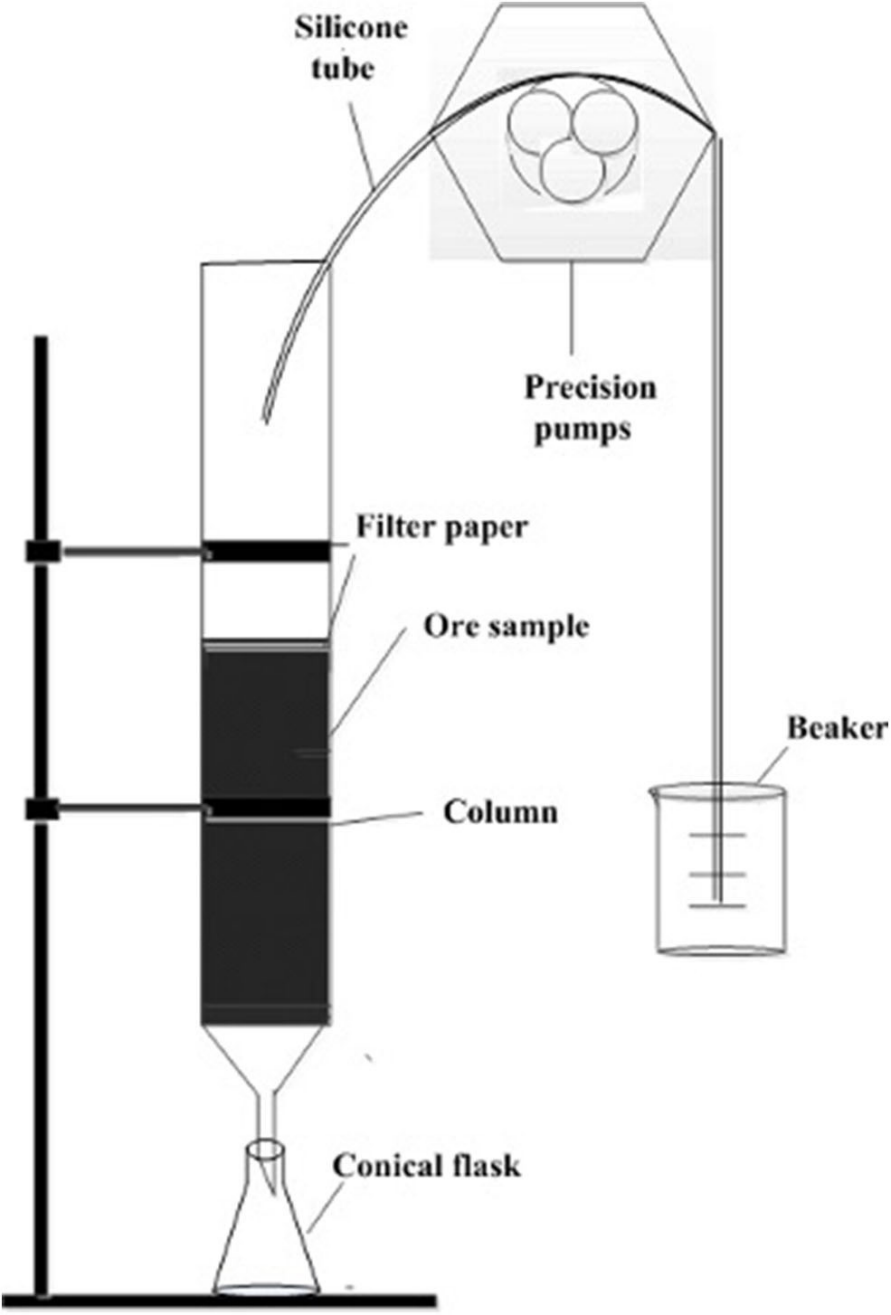
The leaching device of the weathered crust elution-deposited rare earth ore.

In this experiment, the rare earth ore samples were fully mixed evenly and the quartering method was adopted for sampling. Each group of 250 g ore samples were weighed into the leaching column and the surface was lined with filter paper to make the leaching agent seepage even. The formate salts solutions with different concentrations were added into the glass column slowly according to the liquid-solid ratio of 2:1 through the controlled leaching device. By recording the time, the centrifuge tubes were used to receive the mother liquid of rare earth at certain intervals. The rare earth and aluminum contents were analyzed by ICP-MS and the rare earth and aluminum leaching efficiencies were calculated. The leaching device is shown in Figure 3. The rare earth leaching or aluminum efficiency was calculated according to the follow formula:

$$\begin{array}{l} L\left(\%\right)=\frac{\left(C\times V\right)}{W\times C_{S}}\times 100\% \end{array}$$

where L is the rare earth or aluminum leaching efficiency/%, C is the concentration of rare earth or aluminum in leaching lixivium/g/L^−1^, V is the volume of leaching lixivium/L, W is the weight of rare earth ore sample/g, and Cs is the content of rare earth or aluminum in the ionic phase of the ore samples/%.

### The Swelling Experiments of Weathered Crust Elution-Deposited Rare Earth Ore

**Figure 4:**
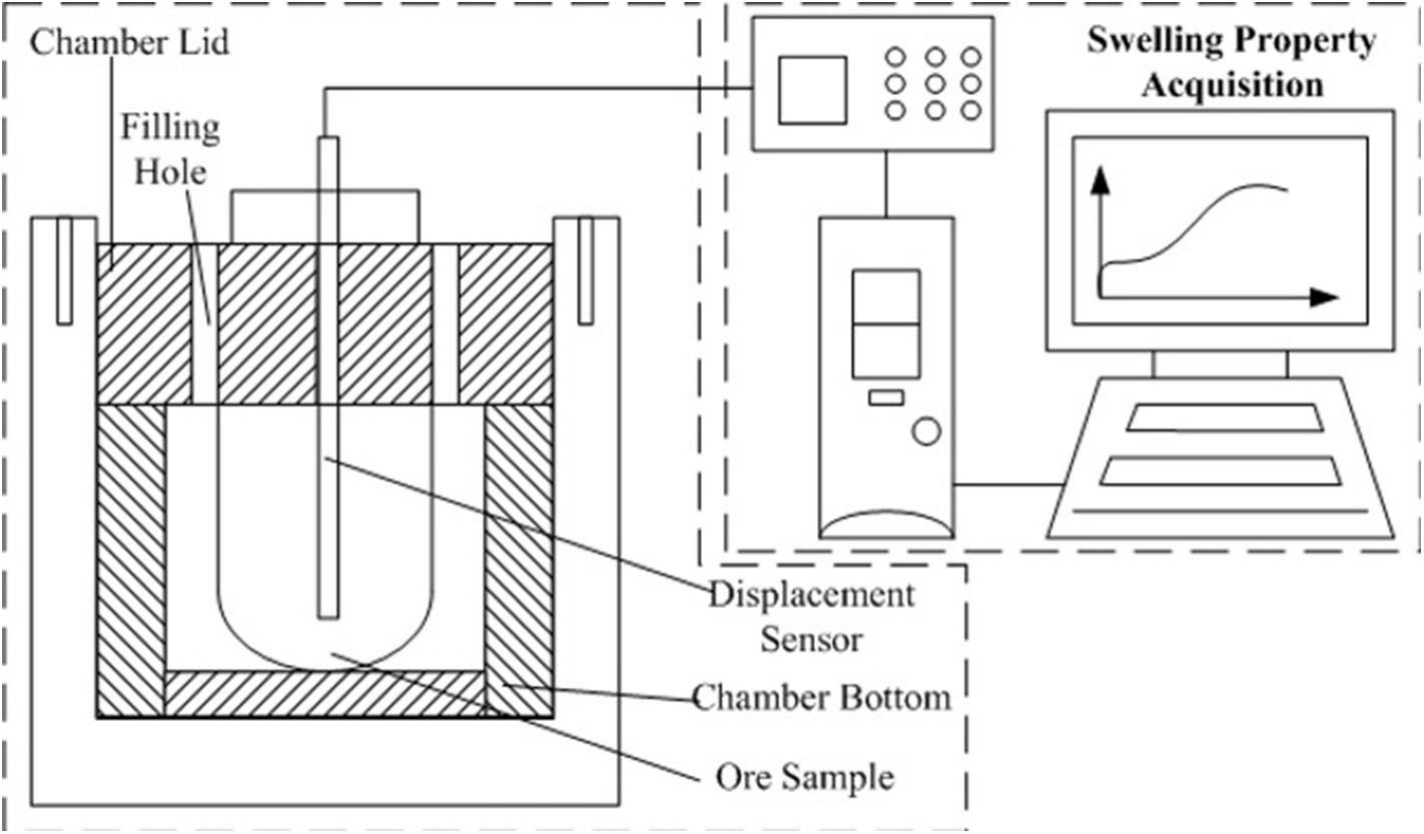
Schematic diagram of PCY intelligent dilatometer.

The swelling of the clay minerals was conducted using a PCY intelligent dilatometer, with the schematic diagram shown in Figure 4.

The swelling ratio of the ore sample was measured by the following equation:

(2) $$\begin{array}{l} \delta =\frac{\Delta H}{H_{0}}\times 100 \end{array}$$

where Δ*H* is the change in height, and *H*_0_ is the initial height of the ore sample.

## Results and Discussions

### Effects of Formate Salts on the Rare Earth Leaching of Weathered Crust Elution-Deposited Rare Earth Ore

**Figure 5:**
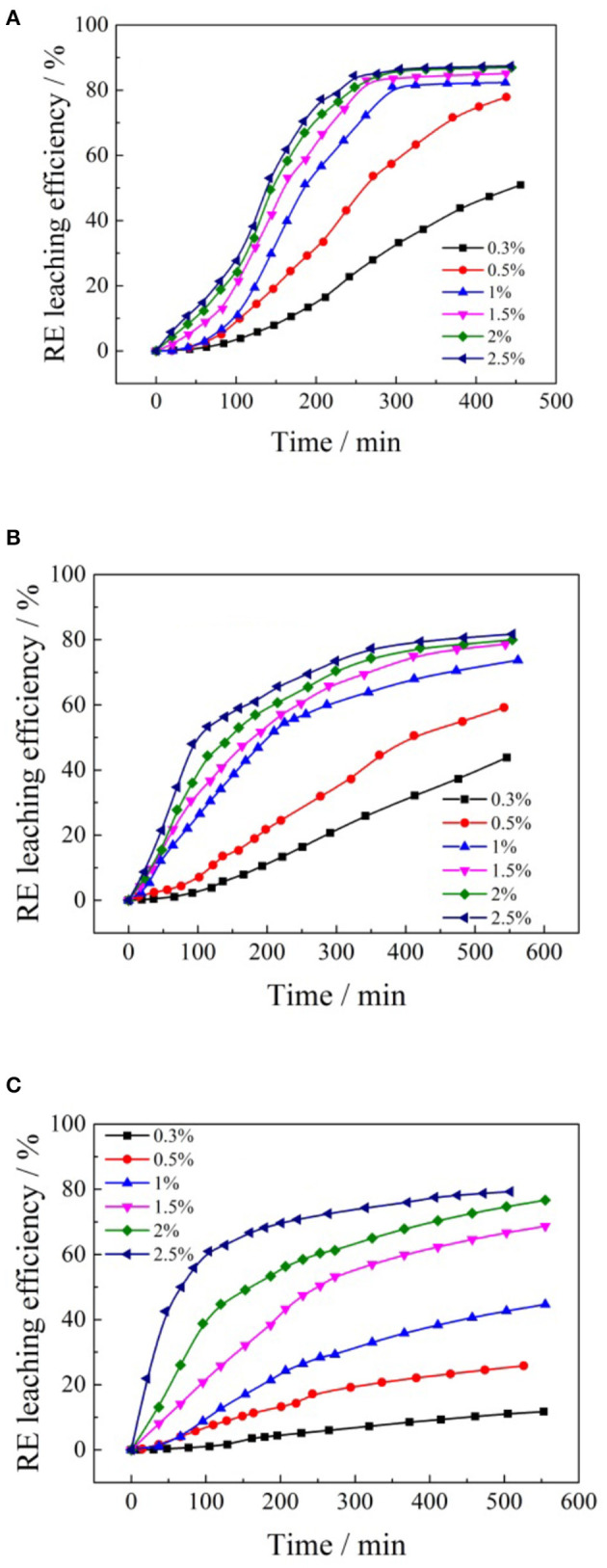
Leaching kinetic curves of different concentrations formate salts for rare earth: **(A)** ammonium formate; **(B)** potassium formate; **(C)** sodium formate.

To investigate the effects of formate salts on the leaching kinetics process of rare earth, various concentrations of ammonium formate, potassium formate, and sodium formate were used as leaching agents, respectively, to undertake leaching experiments under the condition of 2:1 for liquid/solid (mL/g) and pH 5.5–6.0 at room temperature. The results are presented in Figure 5.

**Figure 6:**
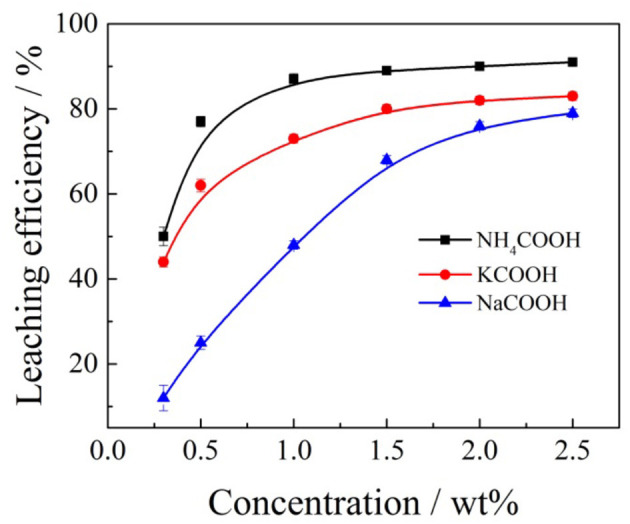
Effects of formate salts concentration on the rare earth leaching efficiency.

It can be seen from Figure 5, with the increase of the concentration in the formate salts, the rare earth leaching rate accelerated. The leaching efficiencies of rare earth increased with the time passed, and the equilibrium was maintained until the maximum leaching efficiencies were reached. As the formate salts solution concentration continued to increase, the leaching rate of rare earth slowed down significantly. With the increase of the concentration of formate salts solution, the leaching rate of rare earth was faster and the time to reach the leaching equilibrium was shorter. The main reason is that the cation (${\text{NH}}_{4}^{+}$, Na^+^, and K^+^) concentration difference between the flow center of the leaching solution (formate salts) and the surface of the clay mineral particle grows larger as the cation concentration in the leaching agent increases. The diffusion ability of the formate salts solution would increase, and the enhancement of the cation concentration will increase the strength of the ion exchange reaction between ${\text{NH}}_{4}^{+}$, K^+^, Na^+^, and RE^3+^, thus speeding up the leaching process (Li et al., 2009; Qiu et al., 2014). In addition, the higher the formate salts solution concentration, the greater its viscosity, and the slower the penetration rate of formate salts into the ore body. The effects of different formate salt concentrations on the leaching efficiencies of rare earth are shown in Figure 6.

According to Figure 6, when ammonium formate, potassium formate, and sodium formate were used as leaching agents, respectively, the rare earth leaching efficiencies gradually increased with the increase of cation (${\text{NH}}_{4}^{+}$, Na^+^, and K^+^) concentration in the leaching agent. When the concentration of ammonium ion, potassium ion, and sodium ion reached 1% wt, 1.5% and 2.5% wt, respectively, the rare earth leaching efficiencies reached a relatively large value, which was 87, 80, and 79%, respectively. It is obvious that the order of the rare earth leaching efficiencies followed ${\text{NH}}_{4}^{+}>$ Na^+^ > K^+^ under the same mass concentrations in the leaching experiments. The cation concentration of formate salts continued to increase, and then maintained a relatively stable trend, and the rare earth leaching efficiencies did not increase significantly. This is mainly due to the fact that formic acid ions can cooperate with rare earth ions well and form complexes with rare earth ions, which can be easily separated and have good water-solubility, which can encourage rare earth ions to leave the clay minerals surface and enter into the leaching solution.

### Effects of Formate Salts on the Aluminum Leaching of Weathered Crust Elution-Deposited Rare Earth Ore

**Figure 7:**
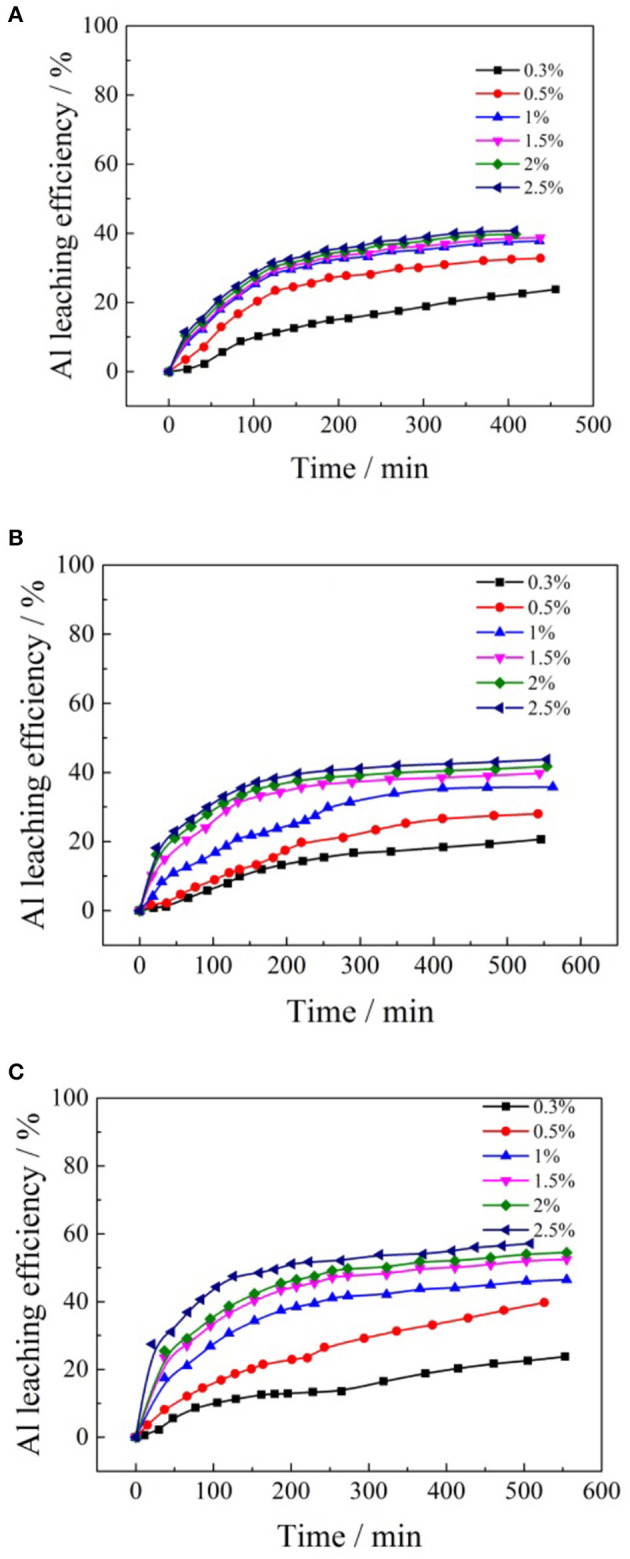
Leaching kinetics of different concentrations formate salts for aluminum: **(A)** ammonium formate; **(B)** potassium formate; **(C)** sodium formate.

To investigate the effects of formate salts on the leaching kinetics process of aluminum, various concentrations of ammonium formate, potassium formate, and sodium formate were used as leaching agents to undertake leaching experiments under the condition of 2:1 for liquid/solid (mL/g) and pH 5.5–6.0 of the leaching agent at room temperature. The results are presented in Figure 7.

**Figure 8:**
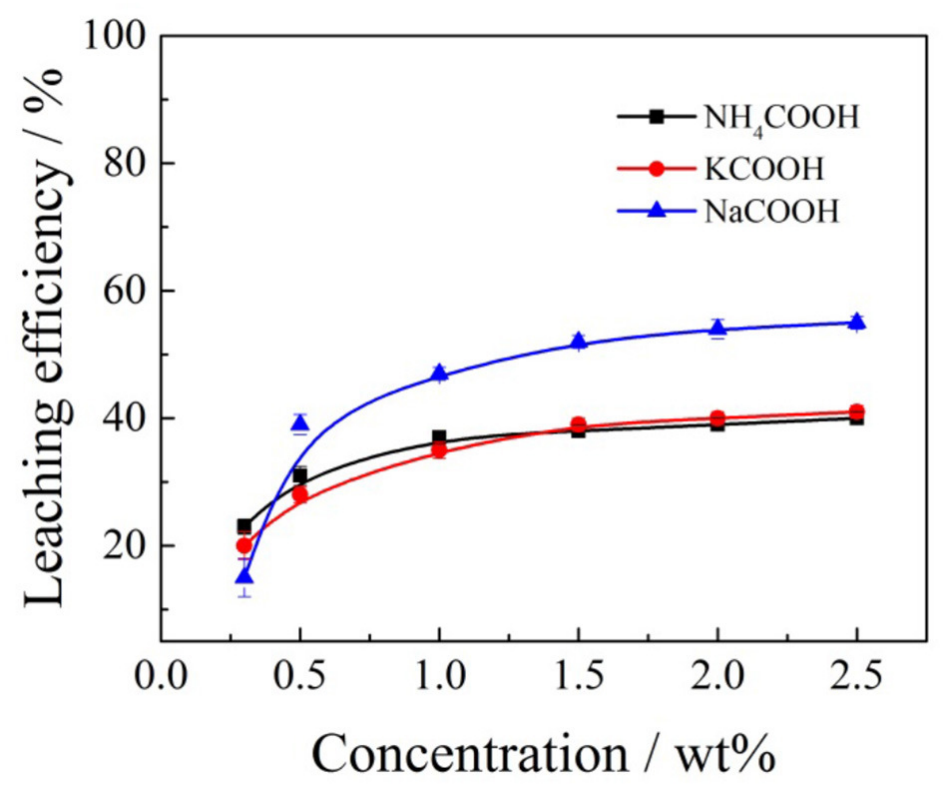
Effects of formate salts concentration on the aluminum leaching efficiency.

It can be seen from Figure 7 that the whole leaching process of aluminum can be divided into two stages. In the initial stage of the ion exchange reaction, the leaching rate of aluminum increases rapidly with time. As the cation concentrations of formate salt increased, the leaching rate of aluminum increased obviously, and the time required to reach the leaching equilibrium decreased. In the same leaching period, more ammonium ions participated in the reaction, and the leaching efficiency of aluminum was improved. The effects of cation concentrations of the formate salts on the leaching efficiencies of aluminum are shown in Figure 8.

According to Figure 8, when ammonium formate, potassium formate, and sodium formate were used as leaching agents, respectively, the aluminum leaching efficiencies gradually increased with the increase of cation (${\text{NH}}_{4}^{+}$, Na^+^, and K^+^) concentration in the formate salts leaching agent. When the concentration of ammonium, potassium, and sodium ions reached 1, 1.5, and 2.5% wt, respectively, the aluminum leaching efficiencies reached a relatively large value, which was 37, 39, and 55%, respectively. The cation concentration of formate salts continued to increase, and then maintained a relatively stable trend. This is the main reason the formate ions in the leaching solutions have stronger polarity to transform ion-exchangeable aluminum into inorganic hydroxyl aluminum and other forms to be retained in the tailing ore samples (Li et al., 2013). Besides, because ammonium formate can ionize more formate ions in the leaching solution, the content of ionic phase aluminum in the lixivium is relatively lower. Therefore, the ammonium formate shows a better inhibiting effect on the aluminum. The impurity aluminum in the lixivium is thus reduced conspicuously, which will be beneficial in improving the efficiency and reducing the cost of the following purification of impurities and the purity of the final production.

### Effects of Formate Salts on the Swelling of Clay Minerals in the Weathered Crust Elution-Deposited Rare Earth Ores

**Figure 9:**
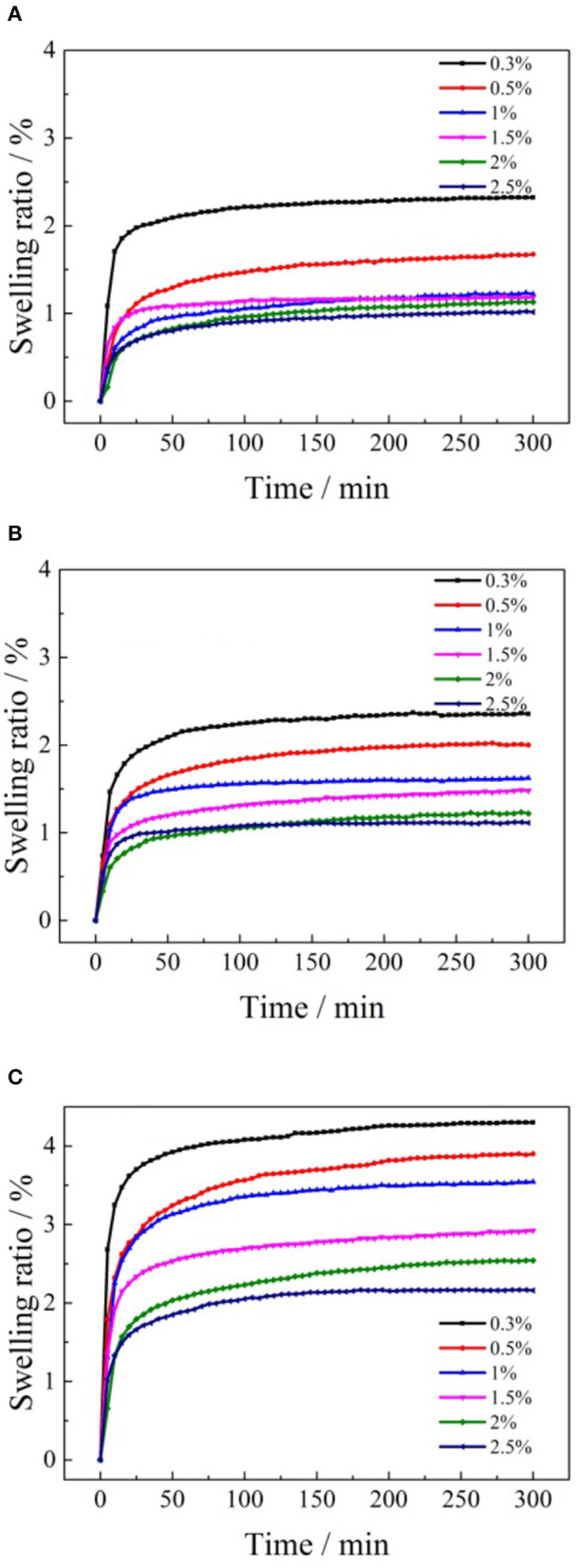
Swelling kinetic curves of ore samples in various formate salt concentrations: **(A)** ammonium formate; **(B)** potassium formate; **(C)** sodium formate.

To understand the effects of the formate salt concentrations on the swelling of the clay minerals, the ore samples were subjected to various concentrations of ammonium formate, potassium formate, and sodium formate. Swelling kinetic curves were constructed using the experimental data, and the results are displayed in Figure 9.

**Figure 10:**
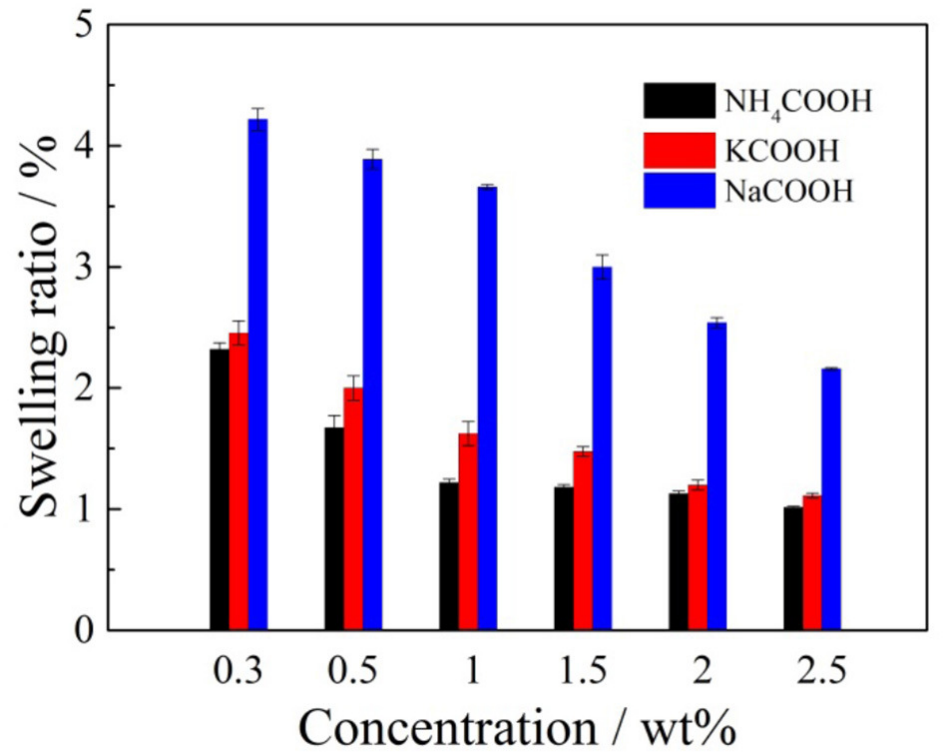
Effects of formate salts on the swelling ratio of clay minerals.

As shown in Figure 9, the clay minerals of the ore sample expanded rapidly after it made contact with the formate solutions of different concentrations. And the swelling ratio of the clay minerals reached a stable equilibrium state within 50 min. After that, the linear expansion rate of the clay minerals remained almost unchanged in the following stage. The effects of cation concentrations of the formate salts on the final swelling ratio of clay minerals in the weathered crust elution-deposited rare earth ore are shown in Figure 10.

According to Figure 10, when ammonium formate, potassium formate, and sodium formate were used to immerse the ore samples to investigate the swelling properties, it was obvious that the swelling ratio decreased gradually with the enhancement of the formate salt concentration. Under the same cation concentrations of the formate salts, the order of the inhibiting swelling ability followed: formate ammonium > formate potassium > formate sodium. The ability of the formate salts to inhibit the hydration of clay minerals in the weathered crust elution-deposited rare earth ore is often related to the cation radius and hydration cation radius in the electrolyte solution (Berrin et al., 2006). The cation radius and hydration cation radius are listed in Table 2.

**Table 2:** Cation radius and hydration cation radius.

**Cations (M^**+**^)**	**${\text{NH}}_{4}^{+}$**	**K^**+**^**	**Na^**+**^**
Cation radius (nm)	0.143	0.133	0.098
Hydration cation radius (nm)	0.532	0.537	0.790
Ion exchange ability	1	2	3

According to Table 2, comparing the cation radius and hydration cation radius of ${\text{NH}}_{4}^{+}$, K^+^, and Na^+^, the larger the cation radius is, the weaker the hydration ability is, and the smaller the hydration radius is, the stronger the adsorption ability is, and the easier it is to enter the layer of the clay mineral to decrease the swelling effects.

### Swelling Kinetics of Clay Minerals in the Weathered Crust Elution-Deposited Rare Earth Ores With Formate Salts

**Figure 11:**
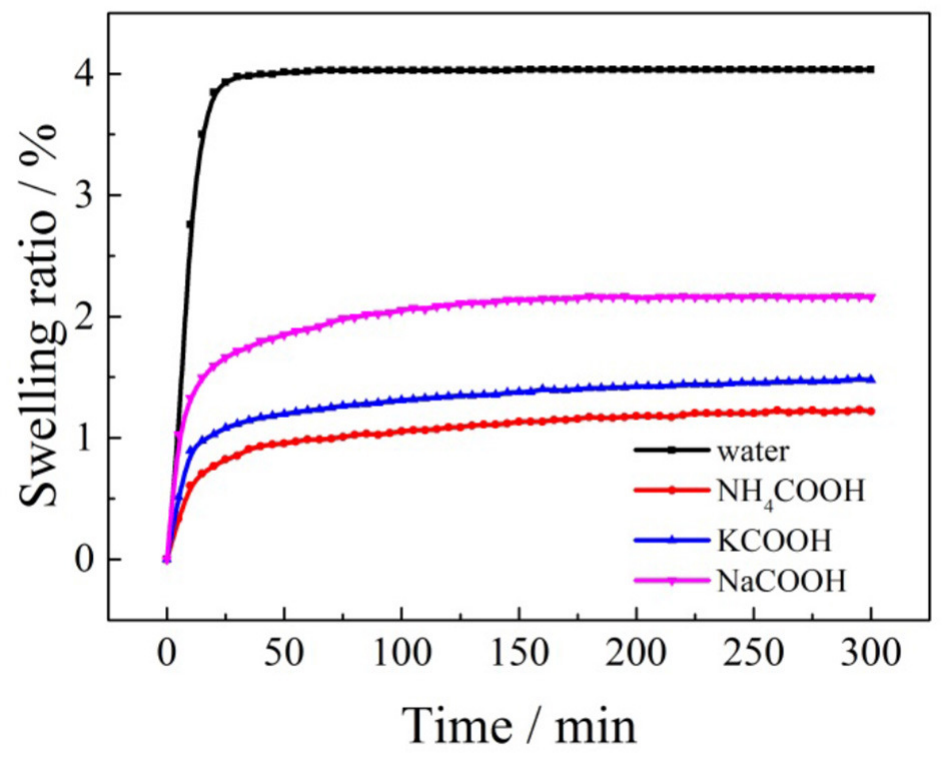
The swelling curves of the formate salts under optimal conditions.

The swelling ratios of the clay mineral samples with formate salts are closely related and changed with time. For example, the swelling kinetic curves of the formate salts under the optimal leaching conditions and distilled water are shown in Figure 11.

According to Figure 11, the swelling process of clay minerals in the weathered crust elution-deposited rare earth ore with formate salts can be divided into two stages: rapid expansion stage and steady state stage (Al-Rawas et al., 1998; Tan and Kong, 2004). In the initial stage, the expansion of the clay mineral sample occurs at the critical surface in contact with the formate salt solution. The clay mineral particles absorb water rapidly, expand at a fast rate, and the expansion rate increases significantly. As the formate salt solution penetrates deeply into the clay mineral and immerses the clay mineral particles totally, the interface between the clay mineral particles and the formate salt solution increases and the ore sample begins to swell completely. Later, after some time, when the clay minerals are completely soaked in the formate salt solution, the water absorption decreases gradually, and the ore sample slowly expands. At this time, the expansion state of the clay mineral enters the second stage, and the expansion rate is greatly reduced and tends to be stable.

**Figure 12:**
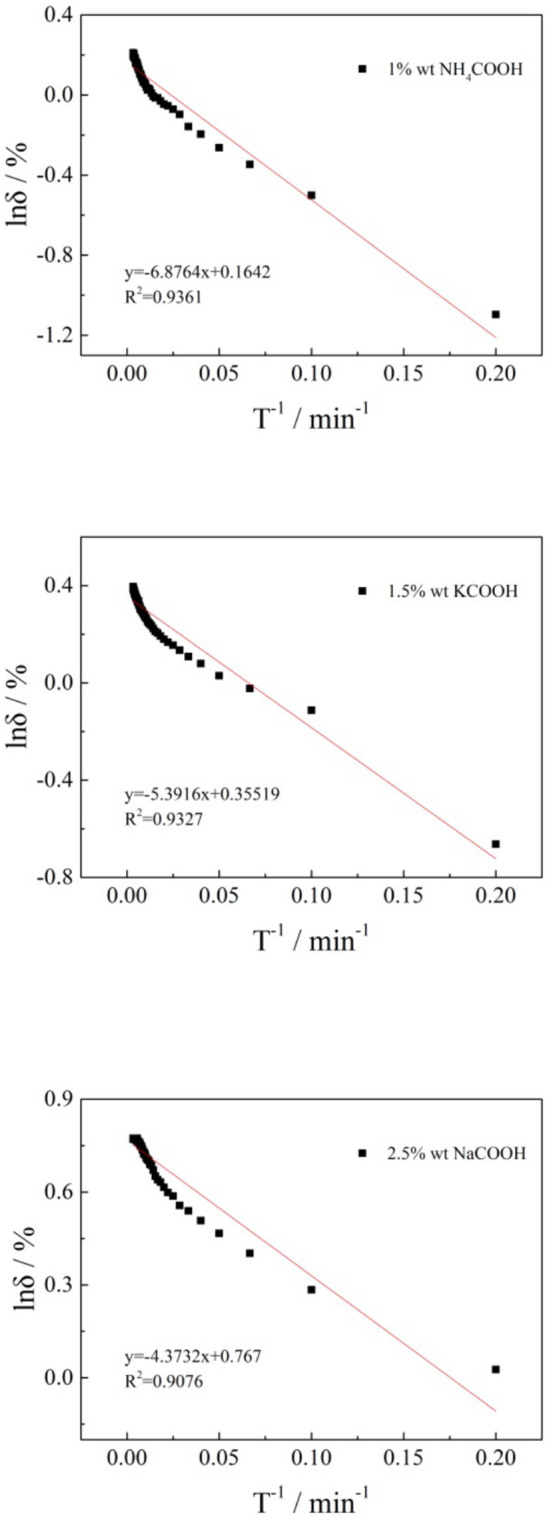
The relation between the natural logarithm of the swelling ratio and the reciprocal of time.

Now take 1 wt% ammonium formate, 1.5 wt% potassium formate, and 2.5 wt% sodium formate as examples to fit their expansion kinetic curves. Figure 12 is shown as the reciprocal of the natural logarithm of the expansion rate to time.

According to Figure 12, there is a certain relationship between the natural logarithm of the clay mineral swelling ratio and the reciprocal of time in the semilog coordinate. In the first part of the expansion, experimental data were concentrated, which reflects the steady stage of the swelling process. In the second half of the expansion, experimental data were relatively separate. By analyzing and calculating the results, the equation of the relationship between the swelling of the clay mineral with 1 wt% ammonium formate, 1.5 wt% potassium formate, and 2.5 wt% sodium formate were presented, respectively: δ = 1.1784∙e^−6.8764/t^, *R*^2^ = 0.9361, δ = 1.4265∙e^−5.3916/t^, *R*^2^ = 0.9327, and δ = 2.1532∙e^−4.3732/t^, *R*^2^ = 0.9076, fitting coefficients >0.9. It shows that the fitting relation was ideal and meets the requirements.

It can be seen from the above results, when formate salts were used as leaching agents to recover the rare earth from the weathered crust elution-deposited rare earth ore, lnδ and t^−1^ showed a good linear relationship which can obtain the relationship between swelling ratio and time: δ = A·e^−B/t^, type A and B are all in the fitting analysis of regression coefficients (Erguler and Ulusay, 2003; Tan and Kong, 2004; Huang et al., 2016).

### Inhibiting Swelling Mechanism of Formate Salts on the Clay Minerals of Weathered Crust Elution-Deposited Rare Earth Ore

**Figure 13:**
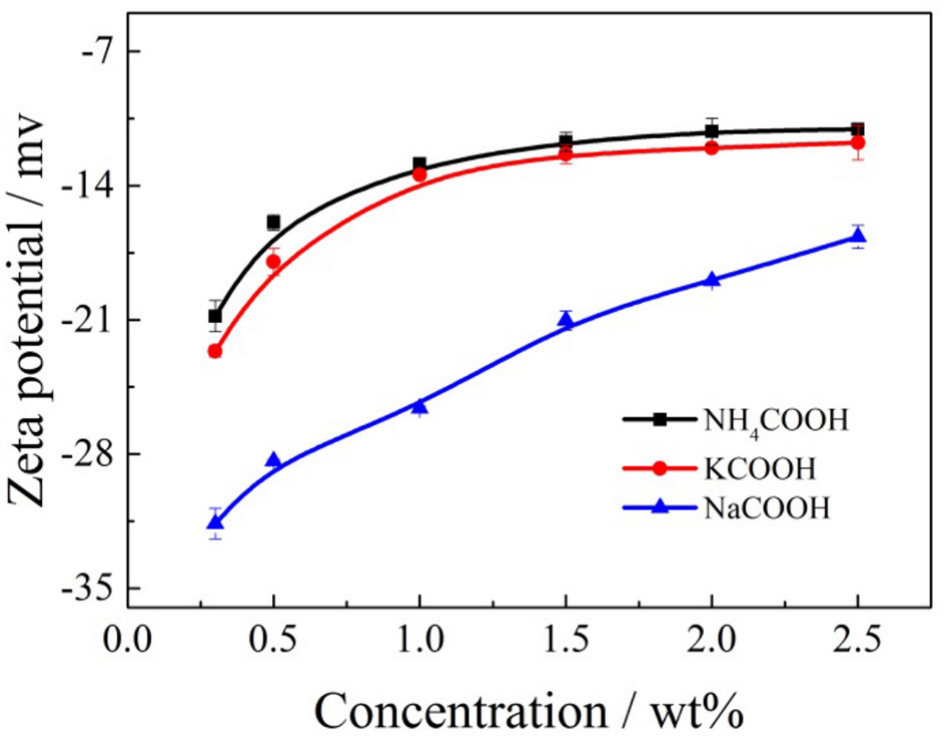
Zeta potential of clay minerals with formate salts.

During the leaching process of weathered crust elution-deposited rare earth ore with formate salts, the utilization of formate salts can effectively inhibit the swelling of clay minerals for preventing landslides and other disasters. The inhibition mechanism of the formate salts on the clay minerals is mainly reflected in the following three aspects (Chen and Wang, 2003; Li et al., 2006; Huang et al., 2013):

1. Charge neutralization. The hydration of the clay minerals is normally relative to the surface potential of the clay minerals when it makes contact with the formate salts solution. The results of the zeta potential of clay minerals with formate salts are presented in Figure 13.

According to Figure 13, with the enhancement of the formate salts concentration, it was evident that the zeta potential between clay mineral particles and formate salts solution increase. The lower the zeta potential, and the higher the effective negative charges of the clay mineral particles, the stronger the ability was of the clay mineral particles to adsorb cations. The layers became thicker, and the flocculation tendency of the clay mineral particles was smaller after they have been soaked in the formate salts, indicating that the clay minerals swell more easily in the low concentration leaching solution. However, with the enhancement of the formate salts concentration, more cations entered the layer, leading to the electronegativity of clay minerals decreasing to some extent, which means the high concentration of formate salt solution can effectively compress the double electric layer. And it finally weakened the hydration of clay minerals to inhibit swelling.

(2) Low activity. Due to the high ion concentration of the formate salts solution, there is less free water and the activity of water is relatively low. According to the activity balance theory, osmotic pressure can reverse the flow of water in rare earth ores. Such reverse osmosis reduces the net flow of water from the formate salts solution to the clay minerals, resulting in the decrease of hydration of the clay minerals and an increase in the strength and actual stress of the capillary pressure, which is beneficial to rare earth ore stability.
(3) The viscosities of formate salts solution are high, making it difficult for leaching solutions to enter the formation. With relatively high salinity and low surface tension, the positive charge on the end surface of clay mineral particles and HCOO– attracts each other, forming a barrier between HCOO– and water to prevent the hydration of clay minerals and stabilize the rare earth ore. Meanwhile, HCOO– and water molecules can form hydrogen bonds and have a strong binding ability to free water, making the filtrate viscosity of formate salts solution high and making it difficult to enter the interlayer of the clay minerals.

## Conclusion

Rare earth is a significant resource all over the world. To recover the rare earth with high efficiency safety, formate salts were used as leaching agents for recovering the rare earth elements. Meanwhile, the swelling proprieties of clay minerals with formate salts were investigated and the anti-swelling mechanism was analyzed. The following conclusions have been drawn:

The rare earth leaching efficiency can reach 87% when 1 wt% ammonium formate was used to leach the rare earth ore, while the leaching efficiency of aluminum was 37%. Besides, at the optimal concentration of 1.5 and 2.5 wt% of potassium formate and sodium formate, the leaching efficiencies of rare earth were 80 and 79%, respectively. Considering the cation radius and hydration cation radius of ${\text{NH}}_{4}^{+}$, K^+^, and Na^+^, the effects of inhibiting the swelling of clay minerals follow the order: ammonium formate > potassium formate > sodium formate. The swelling process of clay minerals has a strong dependence on time. The relationship between the swelling ratio of clay minerals and time is represented by the swelling kinetic equations: formate ammonium δ = 1.1784·e^−6.8764/t^, *R*^2^ = 0.9361, the formate potassium δ = 1.4265·e^−5.3916/t^, *R*^2^ = 0.9327, and the formate sodium δ = 2.1532·e^−4.3732/t^, *R*^2^ = 0.9076. The fitting coefficients are all >0.9, indicating that the relationship between the swelling ratio of clay minerals and time is in agreement with the equation δ = A·e^B/t^ in the leaching process with formate salts.

The result of this research could provide a theoretic guide to the application of formate salts in the industry.

## Data Availability Statement

The raw data supporting the conclusions of this article will be made available by the authors, without undue reservation.

## Author Contributions

ZZ and RC designed the project. ZC performed the experiments. ZZ and ZC analyzed the data. ZC, ZZ, and RC wrote the manuscript. All authors contributed to the article and approved the submitted version.

## Conflict of Interest

The authors declare that the research was conducted in the absence of any commercial or financial relationships that could be construed as a potential conflict of interest.
